# Nitrogen-vacancy centers in diamond for nanoscale magnetic resonance imaging applications

**DOI:** 10.3762/bjnano.10.207

**Published:** 2019-11-04

**Authors:** Alberto Boretti, Lorenzo Rosa, Jonathan Blackledge, Stefania Castelletto

**Affiliations:** 1 Department of Mechanical Engineering, College of Engineering, Prince Mohammad Bin Fahd University, Al Khobar, Saudi Arabia; 2 Faculty of Applied Sciences, Ton Duc Thang University, Ho Chi Minh City, Vietnam; 3 Department of Engineering “Enzo Ferrari”, University of Modena and Reggio Emilia, Modena, Italy; 4 Applied Plasmonics Lab, Centre for Micro-Photonics, Swinburne University of Technology, Hawthorn, Victoria, Australia; 5 School of Electrical and Electronic Engineering, Technological University Dublin, Ireland; 6 Faculty of Science and Technology, University of Wales, Wrexham, United Kingdom; 7 Department of Computer Science, University of Western Cape, Cape Town, South Africa; 8 School of Engineering, RMIT University, Bundoora, Victoria, Australia

**Keywords:** nanodiamonds, nanoscale magnetic resonance imaging (nano-MRI), nitrogen-vacancy center, optically detected magnetic resonance

## Abstract

The nitrogen-vacancy (NV) center is a point defect in diamond with unique properties for use in ultra-sensitive, high-resolution magnetometry. One of the most interesting and challenging applications is nanoscale magnetic resonance imaging (nano-MRI). While many review papers have covered other NV centers in diamond applications, there is no survey targeting the specific development of nano-MRI devices based on NV centers in diamond. Several different nano-MRI methods based on NV centers have been proposed with the goal of improving the spatial and temporal resolution, but without any coordinated effort. After summarizing the main NV magnetic imaging methods, this review presents a survey of the latest advances in NV center nano-MRI.

## Review

### Introduction

Spin echoes and free induction decays were first detected in 1950 [1] and the first nuclear magnetic resonance (NMR) spectrum was reported in 1952 [2]. The first NMR image followed about two decades later, in 1973 [3]. It was not until 1977 that the first human magnetic resonance (MR) images were published [4]. The last few decades have seen the consolidation of MRI as a medical technique, with the purpose of imaging soft body tissues and organs through the excitation of their atomic nuclei with high-frequency radio pulses and the measurement of the response in a strong magnetic field. Recent research has included using MRI for nanoscale imaging, enabling image resolution on the molecular or even the atomic scale. This has given rise to the investigation of nanoscale magnetic resonance imaging (nano-MRI) [5].

Different nano-MRI technologies have been proposed that are based on different sensors. Some of these technologies use the nitrogen-vacancy (NV) centers in diamond as sensors. The NV centers in diamond are one example of a sensor for nano-MRI. Optical measurements with NV centers combined with electron paramagnetic resonance (EPR) were established at the end of the 1970s [6], although it was only in 1991 that EPR was also observed without illumination [7]. The characterization of single NV centers became popular at the end of the 1990s. It was demonstrated that the fluorescence of single NV centers can be detected by room-temperature fluorescence microscopy and that the defect shows perfect photostability [8]. Room-temperature optically detected MR (ODMR) with NV centers was also demonstrated [8].

Conventional MRI makes uses of contrast agents to enhance sensitivity and resolution. In principle, the combination of magnetic and radio-frequency excitation in MRI techniques allows for resolution enhancement both spatially and temporally, while producing deep tissue imaging with outstanding contrast, enabling anatomical and functional observation at the same time. Contrast agents are chemical substances used to enhance and improve the quality of the MRI images. The ferromagnetic or paramagnetic nature of a contrast agent determines the positive or negative imaging contrast for resolution as fine as cell clusters. The more precise the imaging that is required, the higher is the dose of the contrast medium that is necessary. This poses a question of possible toxicity that must be adequately considered and ultimately constitutes a key limitation to the deployment of this technique in clinical practice [9].

Nanoscale MRI or nano-MRI is a novel technique aiming to improve MRI resolution to the nanometer scale (from current values of tens of micrometers), which would enable the measurement of the MR of a single biomolecule. MRI imaging can visualize internal tissues in vivo, and functional MRI (fMRI) can map brain activity with millimeter-scale resolution, which is a significant improvement for clinical diagnosis [10]. This application of nano-MRI is being actively developed with the aim of reaching nanometer-scale resolution, which would allow for single-molecule resolution and extend the imaging techniques to molecular biology. Magnetic imaging is fundamental for exploring chemical–physical magnetic processes and expanding the capacity of magnetic data storage units, enabling high-resolution, real-time imaging beyond the limitations of current approaches based on the magneto-optic Kerr effect in X-ray and electron microscopy [11].

Nano-MRI techniques studied in current research endeavors include several quantum mechanical and nanotechnological approaches, such as optically detected MR (ODMR) using NV centers and MR force microscopy (MRFM). Among the other approaches, magnetic dipole interaction is a new way to replace magnetic induction to allow for nanoscale MRI detection and has delivered promising results in the employment of spin sensors based on atomic-scale diamond impurities. The use of diamond NV centers with nano-MRI has allowed for in vivo imaging on the single-biomolecular scale at room temperature [12].

Other approaches not based on NV centers are evolving at the same pace as methods based on NV centers. For example, a new method for high-resolution nano-MRI coupling high spin sensitivity of nanowire-based MR detection with high-spectral-resolution NMR spectroscopy is presented in [13]. In terms of future commercial products that permit nano-MRI to be undertaken at hospitals and for medical research, it will inevitably be required that they be produced in a way that secures the best trade-off between cost and performance.

Nano-MRI employing nuclear spins is limited by the spin–lattice relaxation time. This time is longer in singlet states. It affects the chance of using weak spin–spin interactions and hyperpolarized media. The symmetry mismatch between singlet and triplet states prevents interaction so that singlet relaxation can only be mediated by higher-order weak processes, making use of adjacent spins which give the same difference bars as radio frequency (RF) access to singlet states. M2S and spin-lock induced crossing (SLIC) [14] are examples of pulse sequences to circumvent this limitation, transferring the polarization of the triplet state by coupling near-equivalent spins depending on spectroscopic features, namely, the *J* coupling parameter and the chemical shift differences. *J* coupling measurements as low as 10 MHz difference have been experimentally shown [15], reflecting the syn- to anti-geometry discrimination ability to resolve protein structures. Since protonic spin lifetimes are on the scale of seconds, long-range detection is limited by the coupling resolution (on the order of 100 MHz), when long-range (up to five or more bonds away) weaker couplings can be detected by transferring to the singlet state.

Magnetic nanoparticles (MNPs) can act as contrast agents [16] to improve the imaging of tumors in specific cancer MRI. Multimodal imaging has been recently made possible by functionalizing the particle surface with biocompatible chemicals, where surface coverings (such as the coronas of proteins) are fundamental to making nanoprobes biocompatible. However, this promising field still needs to overcome challenges to deliver its full potential.

Solid chemical shifts have been measured in 2D with 1 µm accuracy by MR force microscopy [17], a technique that is readily extendable to 3D imaging by exploiting several spectral lines. Fourier/Hadamard transform techniques allow frequency–space multiplexing for faster measurement, where spatial information is recovered through Hadamard encoding and quadrature detection.

Hyperpolarization is a novel functional medical imaging technique that can dramatically increase the signal-to-noise ratio (SNR) in traditional MRI. The method is being applied to small injectable endogenous molecules, which can be used to monitor transient in vivo metabolic events in real time. Among all methodologies, the emergence of hyperpolarized ^13^C-labeled probes (such as ^13^C pyruvate) has best enabled the monitoring of core cellular metabolic events. Hyperpolarized molecular compounds are obtained, for example, from carbon-13-containing molecules. This is done by transferring the electron spin polarization to the ^13^C nuclei at cryogenic temperatures using dynamic nuclear polarization (DNP). This process is followed by rapid dissolution to create an injectable solution in the human body. This procedure can provide up to a 10,000-fold increase in the signal compared to typical thermal polarization conditions. The ^13^C-labeled probes must be well-suited for medical applications and must undergo the hyperpolarization process.

Some methods are proposed in [18], albeit not yet applied in biomedical samples, using nanodiamonds (NDs) as a contrast agent to improve the SNR in conventional MRI. While NDs have been recently applied as theranostic platforms, due to their biocompatibility and low toxicity compared to other nanomaterials, the concentration of ^13^C nuclear spins is diluted (1.1%) in the diamond unless they are enriched with an ^13^C isotope during growth, which is an expensive process. Used in bulk, a high-purity diamond can exhibit ^13^C T1 (spin–lattice relaxation) times of many hours. This is an advantage compared to other liquid-phase compounds as hyperpolarized ^13^C spins usually relax on timescales of T1 ≈60 s to thermal equilibrium. In [18], synthetic, inexpensive, commercial ND with a diameter ranging from a micrometer to 25 nm were hyperpolarized. The large-sized particles were hyperpolarized at 25 mK using the brute force polarization method based on the application of a high magnetic field (4T) to increase the Boltzmann population difference in the nuclear spins. In this case, the spin system thermalizes (loses polarization) on timescales of 53 min. DNP was also used at 4 K and a nuclear polarization of 8% was achieved in larger micrometer-sized diamonds. However, the spin relaxation time was not increased. Hyperpolarization at room temperature for 350 nm NDs in water provided an enhanced ^13^C nuclear spin resonance signal, with relaxation times of several minutes. In terms of relaxation times, these results are not enough for polarization transfer, which is necessary to enable the application of NDs in standard MRI technology.

In this context, in [19], another approach for the hyperpolarization of NDs was pursued. The Overhauser effect is used which is a proton–electron polarization transfer technique. The process transfers the spin polarization from paramagnetic impurities within NDs and the surfaces to ^1^H spins in the surrounding water solution (cross-polarization sequences). This is known as Overhauser-enhanced MRI (OMRI) and the presence of NDs in the solution permits the enhancement of the ^1^H MRI signal that is readily dependent on the ND concentration. NDs are a source of polarized electron spins attributed mostly to a P1 center (nitrogen substitutional) and unpaired electrons at the nanoparticle surface, which are detectable by conventional EPR methods. The procedure involves an AC RF magnetic field in resonance with the EPR spin frequency, driving the ND electron spin polarization which is then transferred to the interacting ^1^H nuclei in the water containing the ND. ^1^H nuclei resonance is then detected by using a standard inductive NMR technique. An enhancement in the detection of ^1^H of −4 over the thermal ^1^H spectrum is achieved. This enhancement is mostly in 125 nm NDs rather than in 18 nm, and it is larger for higher concentrations. However, the relaxation time is shorter at higher ND concentrations. This is not surprising as spin impurities reduce spin relaxation times. Furthermore, high-pressure high-temperature (HPHT) NDs provide a good enhancement while natural diamonds are not useful with this technique due to the low concentration of spin impurities. Vials with deionized water and NDs in solution were imaged using ultra-low-field MRI and OMRI sequences. The difference images from the two methods reveal a higher contrast of the NDs compared to water. Moreover, this method permits a better tracking of NDs in biological samples using OMRI sequences.

### NV-center quantum sensing

There are different concepts associated with NV center sensors for magnetic imaging applications. An NV center is an atomic size point defect in diamond. Standard optical techniques are capable of resolving the photoluminescence signal of a single NV center, the detection of which is facilitated particularly in the negative charge state (NV−). Room temperature manipulation of electron spins at the NV centers by means of magnetic or electric field, microwave or light irradiation, or a combination thereof, allows the generation of sharp resonances in the intensity and wavelength of the NV photoluminescence. The origin of these resonances is interpreted using quantum optics and spin theory, which describe them in terms of spin–orbit interactions and Rabi oscillations. To achieve NV color centers in diamond that are applicable to magnetometry, the reader should refer to the technical information in [20] on the diamond material preparation. Specifically, NV forms in both bulk and ND – this option introduces more opportunity in terms of applications, even if the ND NV magnetic sensing technology is less developed. A review of the superior properties of diamond nanomaterials and the NV centers, as well as their uses in biomedical applications, is given in [21], which includes details on biosensing, bioimaging and drug delivery as well as biocompatibility. The toxicity of diamond nanostructures is also discussed.

Electron spin resonance (ESR) of the NVs themselves is exploited in [22] to achieve single-spin subwavelength resolution in the optical region. Diamond NV centers are used to improve the low SNR and resolution for single-spin images beyond the usual methods of increasing acquisition times and lowering temperatures. MRI using magnetically equivalent NV center spins provides a resolution of less than the wavelength of the readout light, showcasing nanotesla sensitivity and nanometer resolution at room temperature.

The general principles of NV center optical magnetometry are given in [23]. An NV center based single electron magnetometer in a commercial diamond is built under an ODMR microscope in [24]. The optically detected time window is optimized to obtain a better SNR and dynamical decoupling sequences are used to increase the coherence time of the spin sensor by suppressing spin decoherence due to the environment. This increases the sensitivity in the magnetic field amplitude measurements. Dynamical decoupling schemes are based on π pulse trains which command the spin precession abruptly. Recently, dynamical sensitivity control (DYSCO) has been proposed, aiming to provide smooth and analog sensitivity modulation [25]. In this control method, |2π| ambiguities are removed without sacrificing accuracy. An enhancement of the dynamic range by a factor of 4 × 10^3^ is achieved for interrogation times exceeding 2 ms. Optical detection of weak magnetic fields in a spin bath is undertaken in [26] by sensing external magnetic dipoles with fluorescent ND due to the charge dynamics of coupled spins. This provides ultrashort 10 ms acquisition times in sub-nanomolar amounts of Gd spins.

The photobleaching and blinking issues of NV are addressed in [27] by use of fluorescent ND (FND), which has an extremely high NV center concentration. Their lifetimes are longer than fluorescent biomolecules, and the emission can be modulated with a magnetic field giving increased sensitivity. As bioimaging probes, they are versatile, cost-effective and easily functionalized, in addition to being easy to coat with silica for biomedical applications. Background-free imaging, both in vivo and in vitro, is achieved by applying an alternating magnetic field in a traditional fluorescence microscope. As NV centers can be placed much closer to the sample, within a few nanometers of the surface, NV magnetometry has a clear advantage over conventional methods [28–29].

The interaction between the NV center and the sample can be carried out in one of three ways: (1) fabricating the sample on the diamond; (2) placing a nanostructured diamond on the sample surface; (3) holding the NV center probe on a scanner and moving it across the sample surface.

The first approach involves the detection of magnetic fields using near-surface NV centers in bulk diamond. This approach can be further divided into the use of single or an ensemble of NVs for the detection of a few nuclear spins near the surface. The first relies on the longest coherence time of a single NV in ultrapure ^12^C-enriched diamond and in methods to extend the NV coherence time using complex spin manipulation sequences. This approach results in the best achievable sensitivity of magnetic field sensing based on this atomic sensor.

Alternatively, a high concentration of NV centers are formed close to the surface of bulk diamond using delta-doping techniques, and in this case, ensembles of NVs are used to image cells with subcellular resolution, as demonstrated for example in [30]. These examples are also known as diamond magnetometers and are often referred to as vector magnetometry [31], as the orientation and intensity of the magnetic field are extracted.

Currently, an NV ensemble in a single-crystal diamond vector magnetometer [31] can allow for the retrieval of all Cartesian components of a dynamic magnetic field with a bandwidth of 5 Hz to 12.5 kHz for a 50 pT/√Hz magnetic field. The best magnetic field sensitivity currently achievable for a single NV center is of the order of 1 nT·Hz^−1/2^. Higher sensitivity is possible using either NV ensembles <1 pT·Hz^−1/2^ [32] or advanced sensing protocols used in a so-called AC magnetometer [33]. For an ensemble, in bulk experiments, the best broadband sensitivity is 15 pT Hz^−1/2^ over 80 Hz to 2 kHz [34]. Using ensemble AC measurements, the sensitivity of the magnetic field is 0.9 pT·Hz^−1/2^ for a 20 kHz magnetic field [35].

Other NV sensors can be made in an array of NVs in bulk diamond or as nanostructures in bulk diamond, such as nanopillars. This second approach suffers from a lack of scalability in sensor fabrication, so it is rarely used. Scanning NV center probe and scanning magnetic tip magnetic sensing with high spatial resolution [36] have also been proposed. Recent advances in diamond fabrication for scanning tip magnetometry have been achieved by a functionalized ND with NV center magnetic spins scanned by attaching it to the tip of an atomic force microscope (AFM). This can also be achieved by mounting a high-purity diamond nanopillar on an AFM with an NV center placed 10 nm from its end, achieving a sensitivity of 56 nT·Hz^−1/2^, as reported in [37].

Nanodiamond scanning tips currently suffer from a statistical process to integrate the NV in the tips. This limits the applications due to excessive complex and non-scalable fabrication procedures, based on nanoscale manipulations [38]. A more promising approach [39] relies on generating an NV center less than 20 nm below the surface in a diamond nanopillar mounted on a thin platform, typically of less than 1 μm thickness. Coupled with the nanopillar, this diamond film makes a scanning probe when mounted to an AFM head. It is expected that this method can enhance the photoluminescence collected from the NV by a factor of 10.

Finally, ND embedded in a living cell can be directly used as a fluorescence probe and temperature sensor. A review on the use of fluorescent NDs for tracking in living cells is given in [40] and [41]. Later in this review, we discuss cell temperature mapping by ND NV spins. Direct magnetic imaging in cells using NDs has not yet been developed.

Based on the measurements that are performed using NV sensors, NV nano-MRI can be further distinguished, irrespective of whether DC or AC probing methods are implemented. The choice of method is determined by the sensitivity and resolution subject to the required field-of-view. DC methods rely on the application of a fixed small magnetic field and are used to measure constant external magnetic fields. They use, as a read-out, the ODMR of one or more (an ensemble) NVs. DC magnetometry, based on ODMR, can be achieved through the application of a continuous or a pulsed optical and microwave excitation to increase sensitivity. AC magnetometry permits the measurement of variable magnetic fields when pulsed optical excitation and pulsed microwave excitation are used. Various microwave sequences are used such as Rabi oscillation, spin-echo sequences (closer to conventional MRI as it needs a measurement of the spin–spin relaxation T2 time [28]) and universal dynamic decoupling protocols [12,42]. These pulsed sequences are used to increase sensitivity to the point of measuring Larmor frequencies of nuclear spins [12]. Nanoscale MRI frequency encoding is used for coherent control and the site-selective addressing (Rabi oscillations) of 1 × 4 arrays of NV sites separated by ≈15 nm. The order of three NV centers per site have the same orientation, detected by measuring the fluorescence count rates in a standard confocal volume [43].

Stimulated emission depletion (STED) microscopy (see a description of STED in diamond in [44] and [45]) is first obtained to confirm the nanoscale geometry of the sites. An image of four sites is reconstructed by using the NV Fourier magnetic imaging (FMI) technique in k-space [46]. The key ingredient is to use a micro-coil fabricated on the diamond chip to supply electrically tunable magnetic field gradients of ≈0.1 G/nm. In addition, the frequency encoding consists of mapping the positions of spins at various locations onto their resonance frequency, using tunable magnetic field gradients. In fact, DC magnetic gradients attribute a difference ODMR to the four NV sites. By adding to the DC gradient field, frequency-tailored pulse sequences, the method addresses and controls the spins at specific target positions. This technique is derived from conventional biomedical MRI for image slice-selection with millimeter-scale resolution.

Upon applying a DC magnetic field gradient, the Zeeman split between the |±1⟩ states in individual NV centers becomes position-dependent and gets a specific microwave resonance frequency, allowing for individual manipulation. To prove site-selective NV center addressing by frequency encoding, ODMR measurements are performed with a DC electric current induced through a micro-coil to achieve a DC gradient. Four ODMR peaks, corresponding to the four NV sites in the array, are clearly seen. This allows the control of an individual NV site (which holds three identical NVs) using different ODMR frequencies. Site-selective coherent Rabi driving is seen when the frequency is varied to match each site’s ODMR frequency. The NV FMI technique in k-space is then applied to each site by adding an AC magnetic field gradient to super-resolve each site. By synchronizing this gradient with a Hahn echo NV center pulse sequence, the spatial information is encoded on the NV spin phase for each site in “k-space”. The gradient strength, and hence the wavenumber *k*, is gradually increased by stepping up the amplitude of the electrical current supplied to the micro-coil. The four NVs sites are resolved with a spatial resolution ≈30 nm as a single NV site [43]. To improve the spatial resolution, the main challenge is to generate gradients in the magnetic field that can be switched rapidly with respect to the coherence lifetime on the spin that is strong and spatially homogeneous.

NDs are the preferred material for magnetic field imaging in biological samples, but in general, they suffer from a short coherence time T2. This limits their applications in DC and AC magnetometry. It was understood that high-purity ND can accommodate record-long NV T2 times of >60 μs, albeit observed via universal dynamical decoupling [47]. The main decoherence contribution in these NDs is due to nearby nitrogen impurities rather than surface states.

Improved ND NV center electron spin properties were obtained in [48] by a room-temperature near-field etching method. This is based on application of a He–Cd ultraviolet laser (325 nm), which has a longer wavelength than the oxygen molecule absorption edge and can selectively remove the ND surface defects. A decrease in the FWHM of the ODMR spectra close to 15% and an increase in the T2 time of almost 25% are observed, with a maximum T2 of 2 µs. This technique is quite simple and produces better magnetic imaging results using DC magnetic fields in NDs.

### Optically detected magnetic resonance (ODMR) methods

Wide-field microscopy is the method mostly used to implement ODMR-based magnetometry. Often referred to as wide-field magnetometry, it can compare a fluorescence mapping of a sample with its magnetic field mapping. Wide-field magnetometry with NVs allows a wide field-of-view, albeit sometimes with reduced resolution. It has been applied successfully in several fields, mostly in biomedical applications. The pioneering works in NV magnetometry are concerned with proving the underlying principles of nanoscale imaging resolution and magnetic field sensitivity in various contexts. More recently there has been a focus on the improvement of material engineering and diamond magnetometry involving superior designs, as well as applications in a growing variety of fields. In the following sections, we described some of these latest advances and their focus.

An important challenge for improving NV magnetometry is the control of the NV center origin at desired locations in the diamond. The formation of closely spaced NVs to achieve spin-entanglement by direct magnetic dipolar coupling is also a quest for quantum computation and quantum network protocol applications. Nitrogen implantation through lithography-style mask apertures allows the creation of NV centers with high spatial resolution and fine pitch due to the achievable aperture closeness. Such a method used to create an array of NV centers in diamond for magnetometry is discussed in [49], with record spatial localizations on the order of 10 nm in 3D and an inter-NV spacing down to 40 nm (20 nm inter-NV spacing length scale is needed for strong dipolar coupling).

NV center specific ODMR coplanar waveguides (CPWs) are discussed in [50]. The authors achieve bandwidths up to 15.8 GHz, allowing for NV center spin manipulation with external magnetic fields up to 5000 G. The CPW conversion factor is measured by Rabi nutation experiments, ranging from 6.64 to 10.60 G·W^−1/2^ in a frequency band from 0.76 to 17.3 GHz. Broadband CPWs also minimize control pulse distortion, increasing their sharpness and manipulation qualities.

ODMR is combined with pulsed MRI in [51] to obtain ODMR imaging (ODMRI), delivering NV center images at micrometer and nanometer-scale resolution. ODMRI allows for a great advantage, namely, the readout of the spin state simultaneously from the whole sample with high-quality spectroscopic information. This method was further improved in [51] by adding spatially selective spin addressing and manipulation capability, thus improving the spatial resolution. Specific groups of sample spins being selectively manipulated and then the imaging of the entire sample (a fundamental ability for spin-based quantum sensors) are demonstrated in [51].

A key point of NV magnetometry is to set up a correlation with MRI-contrast methods based on conventional MRI. The comparison is achieved only in subcellular imaging due to the scale associated with NV magnetometry. Magnetic imaging probes are important for the analysis of biological and physical systems. Current magnetic imaging techniques make use of magnetic beads to target and follow cells. Direct magnetic imaging of cells has a high detection sensitivity requirement since biological samples have an inherently low natural magnetic background. However, with current magnetic imaging methods, single-cell sensitivity, concurrent with a millimeter field-of-view at the same time cannot be achieved. They either suffer from resolution issues with respect to optical microscopy, have no imaging capability of sub-cellular structures, or involve operating conditions not suitable for living biological samples.

A wide-field NV ensemble magnetometer for optical magnetic imaging of living cells was first used in [30]. A nanometer-scale layer of NV was implanted in arrays close to a bulk diamond chip surface where the magnetotactic bacteria were deposited. Magnetic imaging of living magnetotactic bacteria under room-temperature conditions was achieved with a sub-cellular spatial resolution of 400 nm and a field-of-view of 100 μm. By NV center spin states, vector images of the magnetic field emitted by chains of MNPs, produced by the bacteria placed on the diamond surface, were rapidly reconstructed. These nanoparticle magnetic field maps were correlated with optical images together in the same apparatus. This result provided a new capability for imaging bio-magnetic structures in living cells under room-temperature conditions with high spatial resolution.

Another demonstration of ambient temperature in-cell imaging of biomagnetic nanostructures with resolution down to 400 nm has been given by layering magnetotactic bacteria on diamond with a superficial array of NV centers. Optical detection of quantum spin states allows real-time imaging of bacteria magnetosome chains with a direct correlation to the optical image. Single magnetosomes were thus resolved by the technique on a 100 μm wide field [52].

An alternative approach to fluorescence microscopy is magnetic imaging of cells, labeled or targeted, using MNPs. Single-cell magnetic imaging of immunomagnetically labeled cells was achieved in [53] using a quantum diamond microscope reaching a field-of-view of ≈1 mm^2^, which is a field-of-view two orders of magnitude larger than earlier results of NV imaging on similar samples [30].

Neuronal action potential (AP) magnetic fields have been measured at the macroscale with coarse spatial and/or temporal resolution, using MRI methods and magnetoencephalography for example. This does not supply single-neuron spatial resolution without scalability to functional networks or intact organisms. In [34], AP magnetic sensing has been realized with single-neuron sensitivity and intact organism applicability with an NV quantum diamond microscope under ambient conditions. The method was applied for excised single neurons from marine worm and squid and then to marine worms for extended periods. The key element to making the experiment possible was to bring an NV layer sensor to within ≈10 μm from the specimens, while all other methods (e.g., sensitive superconducting quantum interference devices (SQUIDs)) are limited by distances of millimeters or more from the biological sample. Even with a sensitivity of fT·Hz^−1/2^, the distance does not allow for single-neuron action potential sensitivity.

A key point of NV magnetometry is to set up a correlation with MRI-contrast methods based on conventional MRI. The comparison is typically achieved only in subcellular imaging due to the scale associated with NV magnetometry. A method was developed to correlate the 2D magnetic field measurements obtained by an NV diamond magnetometer to 3D magnetic field images and to finally compare them with conventional MRI contrast and images [54]. A method is proposed to study the connection between subcellular magnetic field imaging using an NV magnetometer and the related MRI contrast. MRI contrast is thus correlated to submicrometer resolution and nanotesla sensitivity magnetic field measurements in biological samples. Molecular-imaging agents such as iron oxide nanoparticles (IONPs) can strongly influence MRI images with their microscale magnetic field gradients produced in the cells and tissues. Macrophage was labeled with 200 nm superparamagnetic IONPs and was internalized. Three-dimensional magnetic field images of the cells were achieved by using NV vector magnetometry. ODMR signals of the four NV orientations in bulk diamond were measured and converted to the orthogonal laboratory reference frame. A procedure for dipole localization in cellular specimens was also developed to reduce the artifact of overlapping dipole effects in the 3D images. This modification of the NV bulk magnetometer was required to reduce photo and thermal (dissipation) damage to the biological sample. For a given minimal and microwave probe power, NV photon emission was enhanced by the optical collection design of the magnetometer, achieved through increasing the NV concentration and by using SiC as a substrate to aid in heat transfer.

After obtaining the 3D maps of the magnetic field in the sample from the magnetic field images, Monte Carlo simulations of the nuclear spin T2 decoherence was performed in lattices of representative cells. This was done to connect micrometer-scale magnetic field measurements to the MRI contrast. The predicted bulk MRI T2 relaxation time was 23.6 ms with an accuracy of 2.8%. Direct experimental MRI measurements of T2 in macrophages prepared for the NV experiment was also performed to determine the accuracy of the reconstruction from the magnetic field images. The distinction between clustered and diffused IONPs was also performed. The NV-based 3D magnetic imaging method was then applied to diagnostic imaging of liver specimens (tissue) from a model of hepatic iron overload in a mouse and of dynamic endocytic uptake of IONPs in live mammalian cells.

Magnetometry based on the electron spin of NV defects in diamond is currently appearing as a platform that is excellently suited for probing condensed matter systems. In addition to room temperature operation for biomedical applications, it can also be used at cryogenic temperatures and has a magnetic field measurement dynamic range from direct current to AC gigahertz bandwidth [33]. As such, NV magnetometry supplies access to static and dynamic magnetic and electronic phenomena at the nanoscale. An NV magnetometer has direct application to the measurements of stray magnetic fields of magnetic samples. Magneto-optic sensor arrays can be fabricated from diamond NV centers and employed to image thin ferromagnetic films. The accuracy obtained is sub-micrometer over surfaces as wide as 100 × 100 µm^2^ and the imaging speed is fast enough to obtain a real-time video of the evolution of stray magnetic patterns. It is not necessary to supply a microwave signal to detect the spin status, which can be read optically by spin relaxation contrast. The sensitivity can thus be improved to less than a microtesla with a subwavelength spatial resolution and sub-millisecond time resolution. This enables the wide-field microscopy of a domain wall with skyrmion dynamics and allows imaging of metal spins via the Hall effect [55].

NV center wide-field microscopy was applied in [11] to characterize and image magnetic samples; sample thin ferromagnetic films were used to map and image their sub-micrometer stray magnetic field patterns by using an array of NV center spins. Using ODMR, wide-field magnetic imaging over an area of 100 × 100 μm^2^ with sub-micrometer spatial resolution (440 nm) and a temporal resolution of 20 ms and 1.5 μT·Hz^−1/2^ sensitivity could be achieved at room temperature. Without using an applied microwave field, all-optical spin relaxation contrast imaging has also been proposed to develop a conventional spin-relaxation time sequence. This method is particularly relevant to imaging without an external magnetic field, which may negatively affect sample magnetization. NV center spin relaxation contrast also allows a new all-optical imaging approach, applicable in the presence of large off-axis magnetic fields.

Reviews of condensed matter physics applications of the NV magnetometer are considered in [56], specifically those involving magnetic fields generated by spins and currents in solids. The use of an NV magnetometer in solid-state physics is applied to the study of static and dynamic magnetic textures and current distributions. As an example, NV magnetometry is used to probe magnets and superconductors, correlated-electron physics, as well as to explore the current distribution in low-dimensional materials. In addition, the study of static magnetic textures (e.g., domain walls and skyrmions), spin waves in ferromagnets and superconducting vortices, and electrical noise currents in metals have been proved feasible with NV magnetometry. Probing static magnetic textures (i.e., the static spin configuration of a magnetic system) is crucial for developing magnetic devices. This is a challenging task in condensed matter physics because the magnetic field reconstruction is based on stray field measurements, which is an example of an under-constrained inverse problem. The stray magnetic field generated by a planar magnetic texture is determined by the spatial derivatives of the local magnetization. The spatial variations of the magnetization of magnetic materials are related to domain walls arising from the connection between regions with different magnetization orientations, having typical widths of ≈10 nm in ferromagnets. This problem is well suited for an NV magnetometer. In fact, NVs are sensitive to the projection of the magnetic field **B** on the NV spin quantization axis (i.e., the **B** || NV-axis). This is the quantity typically measured in NV magnetometry. As such, the full vector field **B** can be reconstructed by measuring any of its components in a plane at a distance, *d*, from the sample if this part is not parallel to the measurement plane. The measurement of the out-of-plane stray field part, *B_z_* (*x*, *y*; *z* = *d* + *h*), can be easily reconstructed under the assumption of the evanescent-field analog of Huygens’ principle from the measured **B** || NV (*x*, *y*; *z* = *d*). An NV magnetometer has been applied to measure magnetic domains of magnetic skyrmions with nanoscale (10–100 nm) spin textures. This is a promising candidate for magnetic storage, due to the ultrasmall-scale features and low currents involved [56].

NV centers can extract the magnetic power spectral density of a system and can be used as a magnetic noise sensor. This supplies a relationship to be set up between the magnetic noise spectrum to the spin and current fluctuations in a material. As such, it could be used to measure the thermal spin noise in low dimensional materials such as hexagonal BN. Further, its sensitivity is enough to measure stray fields due to, for instance, the current density in graphene flakes or stray fields generated by a vortex in a 100 nm thick superconducting film.

Another application of the NV ensemble magnetometer is reported in [57], where the measurement of the lower (first) critical field of three different superconductors, high-*T*_c_ cuprate YBa_2_Cu_3_O_7_−δ (YBCO), and iron-based superconductors is performed, as the lower critical field is a fundamental parameter to characterize a type-II superconductor. Sample cooling to a target temperature below *T*_c_ is done under zero magnetic field, and then a small magnetic field is delivered while recording ODMR signals in various positions on the sample to evaluate homogeneity. From the ODMR splitting at various temperatures, the magnetization is obtained. The transition temperature to superconductors is measured from the ODMR splitting. The material, in the absence of a magnetic field, is then cooled and brought to the superconducting phase. The lower critical field is obtained from the ODMR splitting by varying the magnetic field applied. In the experiment, the spatial resolution was diffraction-limited and the integration time was 4–10 minutes for each data point used to extract the lower critical magnetic field and the absolute value of the penetration depth. Both can be improved using other protocols in NV magnetometry. These are of some of the benefits of linking superconducting material properties with theoretical models.

An array of NV sensors under the diamond surface were used in [58] for the spatial mapping of band bending, where the NV sensors probe the electric field associated with the surface distribution of space charge density under different diamond surface termination. The technique is useful for applications of band bending measurements in electronic devices which need high control of charge distribution, as in engineering of qubit devices with enhanced quantum coherence, for example.

### Other methods

In addition to nuclear spins in proximity to NV centers, it is also possible to optically detect electron spins, as paramagnetic centers in the diamond lattice or diamond surface radicals, via their coupling to the NV center. Due to interactions of the electron spins with each other, their detected spectra are often broadened to supply critical chemical information. Cross-relaxation phenomena between NVs and other paramagnetic defects in diamond was used in [59] to detect the presence of the well-known electron spin from the N substitutional centers (P1) and the NV neutral charge state. Both of these paramagnetism defects cannot be optically detected. Only the negative charge state of NV, due to its metastable state properties, permits ODMR. However, due to their low spin state, they have been clearly demonstrated by EPR in the presence of a magnetic field and added microwave excitation. In this work, the authors proved that by using NV ODMR, it is possible to show their resonances in the NV cross-relaxation ODMR spectrum for low magnetic fields (a few milliwatts). This technique allows ODMR to replace EPR methods that require high **B**-fields to provide detailed spectra of the detected electron spins for identification of radicals or relaxation centers, with low concentration defect resolution. However, this method needs an ensemble of NVs and it is not clear how it is comparable with other methods that are able to measure dilute electron spin resonance in solids with microwave signal read-outs. In biomedical applications, EPR methods of radicals have sensitivity limitations that typically restrict the imaging resolution to ≈10 µm.

High resolution of electron spin imaging of a target solution of hexaaqua Cu^2+^ complex using an array of NV centers in diamond and the cross-relaxation method was achieved [60]. This EPR method discriminates between electronic spin species by accurately setting the magnetic field to make the NV center resonate with the external target spin. The method has been tested by imaging a diamond chip with an image mask made up of a series of gratings of width ≈500 nm and a pitch of 1 μm. The minimum resolution compares with the microscope diffraction limit, which is ≈305 nm over a field-of-view of 50 × 50 μm^2^ with a spin sensitivity of 104 spins per voxel or ≈100 zmol. This method can enable the development of electron spin resonance with zeptomole sensitivity in chemical sciences.

Super-resolution imaging beyond the diffraction limit using spin defects has recently been developed using NV centers by STED, STED-ODMR, reversible saturable optical fluorescence transitions (SPIN)-RESOLFT, stochastic optical reconstruction microscopy (STORM) ODMR and localization microscopy as reviewed in [45] in both bulk and NDs. Super-resolution spin microscopy is, however, difficult to implement in NDs due to the random orientation of the crystal main axis, and as such, of the magnetization axis of the NVs. Investigations have been carried out on the environment of the NV centers within the same ND to study the quantum properties of NV centers.

A method of spin-manipulated nanoscopy has been set up for nanoscale resolution imaging of collectively blinking NV centers when they are confined within the diffraction-limited region [61]. Collective spontaneous emission in nanomaterials is a common feature and relates to fundamental photophysical properties. Multiple NV emission in 45 nm or less is often subject to collective emission. Using wide-field localization microscopy combined with nanoscale spin manipulation based on a microwave source tuned to the ODMR frequency, two collectively blinking NV centers could be resolved within the diffraction limit. Here, fundamental studies of the coupling of photoluminescence and spin properties of NV in diffraction-limited space are carried out by a super-resolution microscope. In the presence of a single ground state spin transition frequency for all emitters, the external magnetic field is used to split the resonant dips to individually resolve each NV center. Using NV spin properties creating three-level blinking dynamics, two NV centers in the same ND were localized at 23 nm.

NDs can host several paramagnetic point defects and impurities along the diamond surface, which are dark and can broaden the NV spin spectra. These dark defects limit the use of NVs in NDs for magnetic imaging. The composition and spin dynamics of a particle-hosted spin bath are examined in [62] by ODMR inside a 45 nm diamond nanocrystal, revealing nitrogen donors and an unidentified class of paramagnetic centers. Both show a spin lifetime much shorter than that of the NV center spin. By dynamical decoupling and double spin resonance, it is possible to achieve NV center coherence lifetimes comparable to bulk.

Spin-reversible saturable optical fluorescence transition (spin-RESOLFT) microscopy is used in [63] for accurate nanoscale imaging of spatially varying magnetic field patterns with resolution as low as ≈20 nm transverse to the beam (*x*- and *y*-axis), while precision parallel to the beam (*z*-axis) can be pushed down to sub-nanometer values by combining spin-RESOLFT with NV-center NMR measurements from proton spins in a sample facing the diamond. In RESOLFT, the NV center ground-state spins are initialized by a Gaussian laser beam and then spin manipulation occurs as in typical confocal systems. An example of this is the dynamical decoupling of microwave pulse sequences based on spatially selective repolarization via a pulsed green doughnut beam which is introduced before reading out the spin to pinpoint a specific NV center within the sub-diffraction limited area of the Gaussian beam. Additionally, shallow NVs have been used for measuring proton NMR signals that are facing the diamond, with 50 nm transverse resolution and while preserving the proton NMR linewidth. In this case, a sequence of XY8-4 pulses (see modalities for NMR using NV centers below) was employed for sensing the Larmor frequency in a RESOLFT mode for proton spins in immersion oil placed on the diamond surface.

Optical probes are attractive for imaging neuronal action potentials (APs) due to high signal-to-noise. Unfortunately, optical probes can also induce toxicity and can interfere with the network activity of the system during imaging. As NDs have been used to image the APs of single neurons, in [64], a study of the electrophysiological effects of NVs in NDs in primary cortical neurons is reported. Multielectrode array recordings are used across five replicate studies where NDs are added at different concentrations up to 20 μg/mL for a period of 12 to 36 h. No statistically significant difference is seen in neuronal behaviors over 25 neuronal network parameter comparisons. This physiological study motivated the culture of neuronal networks onto a specific glass coverslip with embedded gold microwave resonator for ODMR sensing coated with polydimethylsiloxane using the same culturing procedure as for the physiological study. The ND suspension was dispersed in cell media and then applied at a concentration of 6 μg/mL to the primary cultures while performing a routine change of cell media. ODMR from NVs within the NDs in the neural networks was used for sensing the temperature from thousands of NDs, which were probed simultaneously using a wide-field imaging system. It was seen that at zero magnetic field, the majority of NDs have a crystal field splitting of *D* = 2.87 GHz. By recording ODMR of the NDs at different temperatures a variation of the zero-field splitting *D* was seen, corresponding to a known local temperature variation. The temperature map was then obtained at each location by converting the shift in *D* into temperature, using d*D*/d*t* = −74 kHz/K. This demonstrated the intracellular temperature mapping in primary cortical neurons with a high diffraction-limited spatial resolution. This approach may be of interest for optogenetics, which is a traditional and widely used technique for neuronal stimulation. However, due to NDs strains that may affect the dip in the ODMR signal, the accuracy of the mapping is limited. Developing multifunctional sensors is truly relevant for applications under physiological conditions and for monitoring intracellular processes relevant in biological and medical applications. In this context, hybrid systems using NV sensors with other MRI contrast agents or sensors have been proposed such as iron oxide nanoparticles and paramagnetic gadolinium complexes.

SPIONs are single-domain magnetic systems whose features are used in many technologies, from magnetic information storage to ferrofluids or nanoscale drug-delivery systems and magneto-assisted hyperthermia cancer treatments [65]. Single SPION detection with 10 nm accuracy was shown by bulk diamond NV center magnetometry combining spin relaxation time (T1) and spin dephasing time (T2) Hahn-echo measurements. The formation of hybrid systems between NDs and SPIONs is of growing interest to enhance NV magnetometry in the local nanoenvironment.

A single NV center was functionalized with a SPION by an AFM pick-and-place approach in [66]. It is shown that in the NV-SPION system, the NV spin relaxation time is reduced, while the T2 coherence dephasing time stays the same. By configuring the applied AC magnetic fields, the NV electron spin Rabi oscillation rate decreased, due to a resultant superparamagnetic nanoparticle magnetization at the NV center Larmor frequency. The work shows the effect of coupling single NV to single SPION systems for magnetic sensing. However, its applicability to biological samples is difficult in practice due to the need for multiple NDs coupled to SPIONs.

Paramagnetic gadolinium complexes were attached to NDs with NV centers by properly engineering the diamond surface in [67]. A hybrid nanoscale sensor is constructed that can detect physiological species through a proposed sensing scheme based on NV spin relaxation time measurements. The Gd^3+^ complexes are spin labels attached to a polymer shell covering the ND surface. After activation of a chemical switch due to a local change, which can be monitored by a change in the T1 relaxation time of NV centers, Gd^3+^ complexes are released. The method consists of measuring spin relaxation rates enabling time-dependent recordings of changes in pH or redox potential at a sub-micrometer-length scale. The test was performed in a microfluidic channel that mimics cellular environments. This method can be generalized to other ND hybrid systems, where the release of an attached sensing species on the surface of the ND due to some chemical reaction can be monitored by the spin relaxation time of the NV center. Smaller NDs with long spin relaxation times can be beneficial for this application.

A study of NVs in ND spin properties under a dynamical environment is performed in [68]. NVs in NDs are sensitive to the magnetic, temperature and electric properties of the nanoenvironment. However, a study of the dynamic conditions of the nanoenvironment has not been performed to date. This is important in applications of NV centers in NDs for tracking living cells. It has been shown that the electron spin resonance linewidth of the single NV center broadens to match the rotational diffusion constant of the host ND. When NDs gradually detach from the substrates in an aqueous buffer solution, their ODMR peaks are broadened by 1.8 MHz, which corresponds to the rotational diffusion constant of NDs with a diameter of 11.4 nm from the Einstein–Smoluchowski relation [69–70]. This work can enable the investigation of nanometer-scale fluid mechanics by the measurement of the rotational Brownian motion of single nanoparticles.

Wide-field ODMR magnetometry was used for strain imaging of NV centers in [71], resulting in preferentially aligned polycrystalline diamond along the grain boundaries with a higher spin coherence time as with single-crystal samples. This observation can improve the signal-to-noise of NV sensing applications and can allow the use of polycrystalline diamond with large areas as diamond sensors. The method can also be used to study strain in other materials with optically accessible spin defects or in strain-engineered devices, as well as for mapping externally applied strain in specific materials.

### NV-center nano-MRI schemes

The latest advances in nano-MRI using NV centers are reviewed in this section. This includes NV magnetometry developments to sense external nuclear spins (NV NMR spectroscopy). More general advances in NV center magnetometry methods that may help nano-MRI with NV centers of various systems are also included.

**Figure 1:**
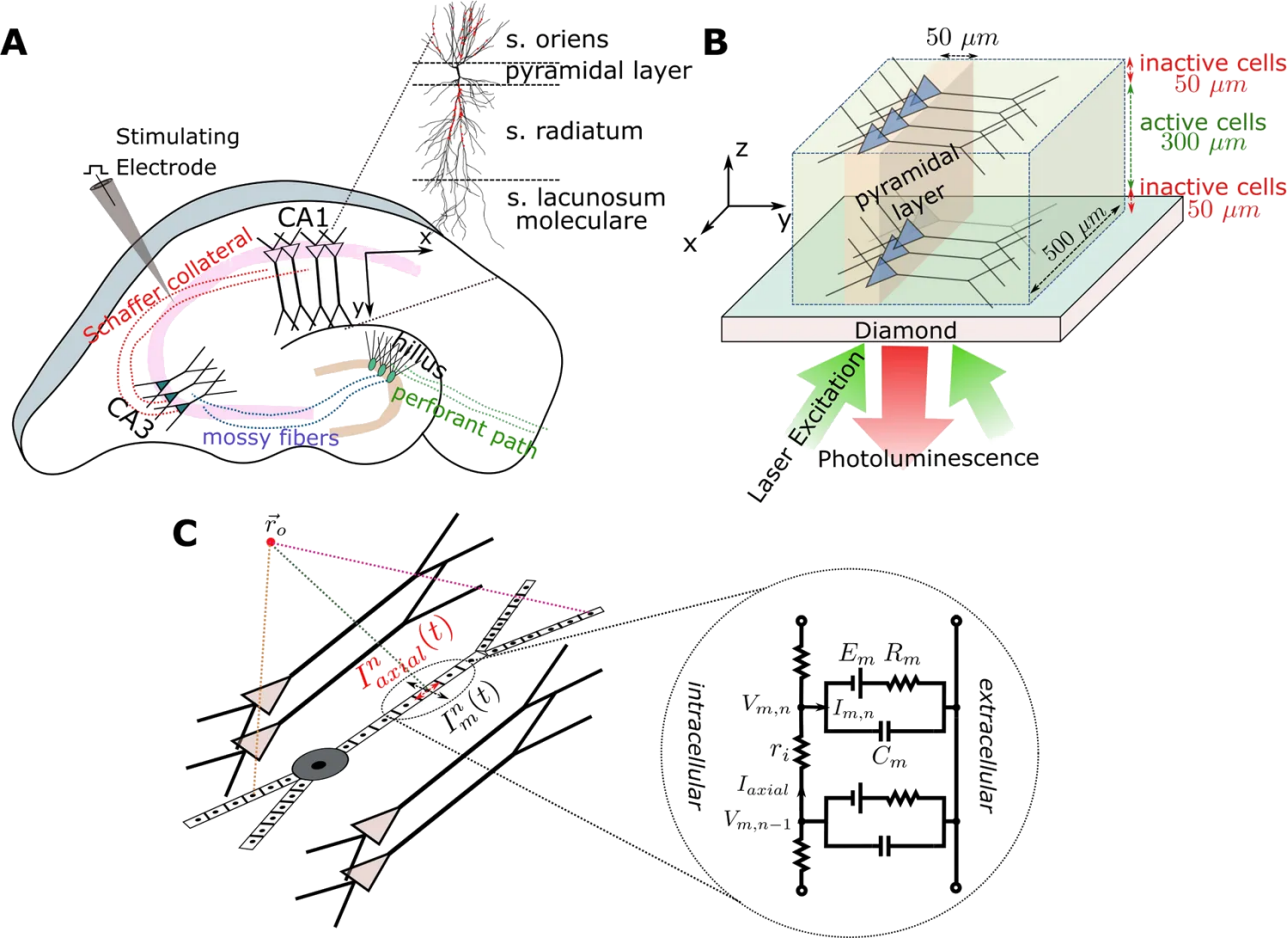
Diamond NV center mediated optomagnetic imaging of brain sample neural activity [72]. Image reproduced with permission from [72], an article licensed under a Creative Commons Attribution 4.0 International License, https://creativecommons.org/licenses/by/4.0/. (A) Trisynaptic path in hippocampus sample. Electrical stimulation of the Schaffer collaterals evokes CA1 area activity which is then recorded. (B) 500 × 500 × 300 µm^3^ simulated CA1 subareas on top of the diamond surface, assuming up to 50 µm distance from the diamond makes neurons dysfunctional. Along *x*- and *z*-axis, the distribution of pyramidal cells is uniform, their soma locations being randomly jittered in a 50 µm wide band along the *y*-axis. Inverted microscope with a camera in place of a photodetector is used to detect the photoluminescence change in NV center layer emission due to neural magnetic fields. (C) Forward modeling scheme pyramidal cell multi-compartment model.

Figure 1 presents the NV center optomagnetic imaging of active networks of neurons in brain samples [72]; a new way of achieving wide-field neural dynamics due to the high sensitivity of NV centers. The investigation of neural activity and the insight in neural information processing mechanisms can thus be achieved by making use of the established method of in vitro brain slice observation, overcoming its inherent limitation in that traditionally it is not possible to detect signals from the whole of the network at the same time. In fact, NV center magnetometry makes it possible to detect the spatial and temporal features of magnetic fields produced by neural activity across the sample, by use of models extracting the main parameters of the neural signals and allows the ready comparison with a single pyramidal cell [72]. This is the new frontier, as NV center magnetometers are being readily improved to image full brain slice activity, while the forefront of research is focusing on the issues of planar cell imaging for single-shot measurement.

Among AC magnetometry techniques, NV Fourier magnetic imaging (FMI) [46] is used besides traditional MRI imaging. Here an AC gradient in the magnetic field (the pulsed magnetic field gradient) based on microcoils brings a phase encoding based on the position in “*k*-space” of the NV center electronic spins, which is then converted to a real space image via conventional Fourier inversion.

The key advantages compared to imaging in the real space is the multiplexed position detection, which enhances the SNR for characteristic NV center densities, supplying a high data acquisition rate, and the simultaneous readout of all NV center signals in the field-of-view. The sequence of FMI pulses relies on a laser initialization pulse, followed by a microwave sequence for dynamical-decoupling spin-state manipulation, an AC magnetic field gradient, and finally, a laser readout pulse.

A key challenge in nanoscale magnetic imaging in NV centers in diamond is to scale up NV spin control to arrays of NV spins. A controlling array of spins address the implementation of nanoscale spin networks and achieve magnetic imaging with a high spatial dynamic range.

**Figure 2:**
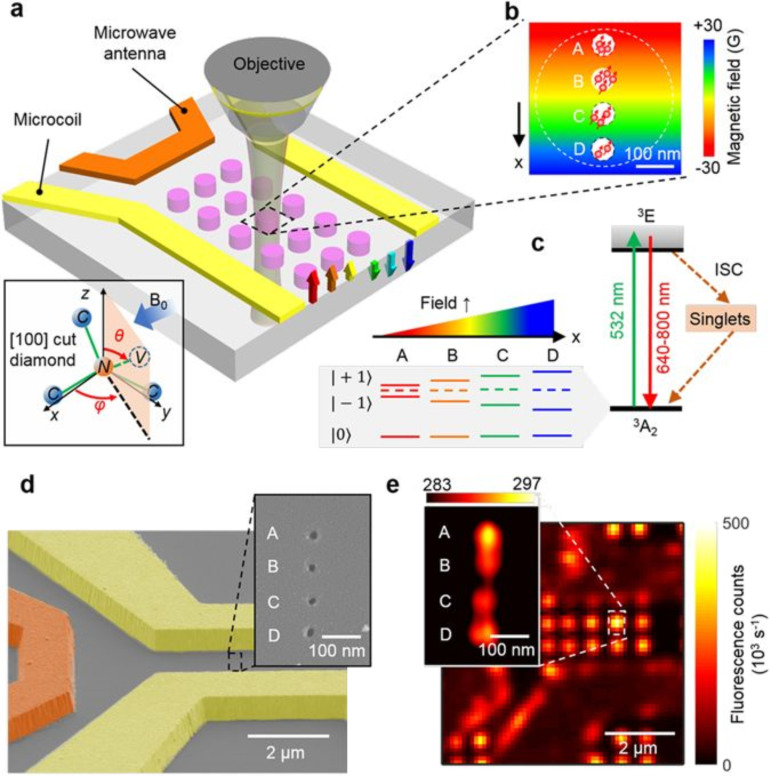
Experimental set-up and characterization of NV centers and the gradient microcoil of [43]. Image reproduced with permission from [43], an article licensed under a Creative Commons Attribution 4.0 International License, https://creativecommons.org/licenses/by/4.0/. (A) Experimental apparatus. A matrix of implantation regions (pink circles) of negative charge NV centers on the diamond is integrated into a scanning confocal microscope. A green laser (532 nm) is used to initialize and read out NV center spin states in a given region and microwaves emitted by an antenna (orange bar) manipulate them. A uniform magnetic bias field of *B*_0_ = 128 G is applied along one range of NV orientations, causing a Zeeman line splitting among |±1⟩ NV center spin states; without a magnetic field they would show a 2.87 GHz frequency shift from the |0⟩ state. An added gradient magnetic field (rainbow-colored arrows) of ≈0.1 G·nm^−1^ is applied by injecting current along a pair of gold wires (gradient microcoil), inducing the Zeeman shift depending on the position. (B) Each region (pink circle in a) holds a 1 × 4 array of NV sites with 60 nm diameter and 100 nm spacing, which undergo interaction that depends on the gradient in the magnetic field. Each site typically holds multiple (≈3 ± 1) NVs of the selected orientation. (C) NV energy-level diagram. A nonradiative intersystem crossing channel exists between the ground (3A2) and excited (3E) states, and the magnetic field gradient causes the NV center spin states |±1⟩ Zeeman splitting to be dependent on their position. Each site has a specific resonance that the microwave generator can be tuned to, allowing for selective detection of each site. (D) Gradient microcoil SEM image on the diamond substrate. The microcoil, represented by yellow pseudo-color, is 1 µm thick and 2 µm wide. (Inset) E-beam resist apertures on PMMA, SEM image of ion implantation mask to create a 1 × 4 array of NV sites. (E) Microcoil and NV center matrix image by scanning confocal microscopy. (Inset) STED image of 1 × 4 array of NV sites with 50 nm resolution. Photon count rate (kilo-counts-per-second) is shown by color table.

The spatial control of an array of four spin sites at the nanoscale has been achieved in [43] by combining a microcoil supplying a DC magnetic field gradient and FMI, as shown in Figure 2. The microcoil replaces the DC magnetic gradient; otherwise achieved by a scanning magnetic tip, and has the relative advantages to improve the bandwidth of gradient switching and space dynamic range. Nanoscale MR frequency encoding is applied in [43] to selectively address positions and coherently control a four-site NV spin array. Positional separation in the array is 100 nm, and every site is made up of multiple NV centers with *a* ≈ 15 nm spacing. Microcoils are built on top of the diamond to inject electrically tunable magnetic field gradients of ≈0.1 G/nm. Site-selective NV center spin manipulation and sensing become possible as the gradient fields and resonant microwave pulses are suitably arranged, allowing for applications such as Rabi oscillations, imaging, and NMR spectroscopy with nanoscale resolution.

**Figure 3:**
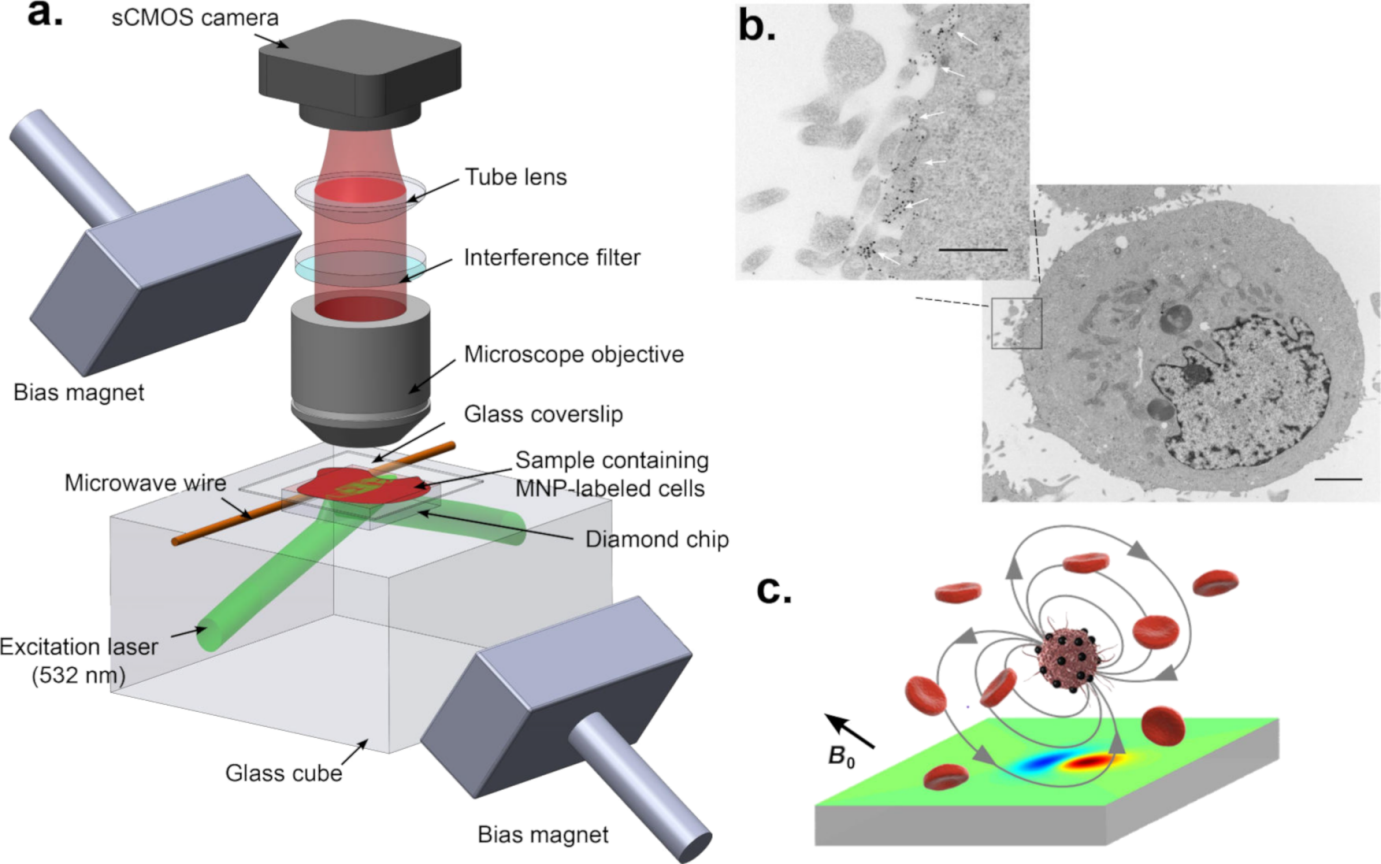
Single-cell magnetic imaging quantum diamond microscope [53]. Image reprinted with permission from [53], copyright 2015, Springer Nature Publishing. (a) Wide-field magnetic imaging microscope based on NV centers in diamond. Immunomagnetically labeled cells are positioned on top of a diamond with a surface layer of NV centers. A 532 nm laser is used to measure the ODMR of the NV electronic spins. An sCMOS camera is used to image the NV fluorescence. The magnetic field projection along one of the [111] diamond axes is measured over a 1 mm × 0.6 mm field-of-view for each imaging pixel. The scheme is adapted from [30] where further details are provided. (b) Electron micrograph of an SKBR3 cell labeled with MNPs conjugated to HER2 antibodies. The black dots on the cell membrane highlighted by the white arrows in the expanded view indicate the magnetic nanoparticles. The main figure scale bar is 2 μm. The inset scale bar is 500 nm. (c) MNP-labeled diagram of a target and surroundings with unlabeled normal blood cells. The magnetic bias field *B*_0_ externally applied is aligned with the diamond [111] axis. This field is used to magnetize the MNP labels. These magnetized nanoparticles produce the field then are imaged by the shallow NV center layer that is close to the surface of the diamond surface to show a dipole-like pattern.

Figure 3 presents the single-cell magnetic imaging quantum diamond microscope of [53]. A bulk diamond microscope is employed to covert the readout of immunomagnetically labeled cells into an image. Correlated magnetic and fluorescence imaging of the sample can be obtained using the NV centers in the diamond. The so constructed diamond microscope can provide single-cell resolution with a field-of-view of ≈1 mm^2^. In the sample application, Glenn et al. [53] successfully measured cancer biomarkers expressed by rare tumor cells within a large population of healthy cells.

The quantum diamond microscope relies on a bulk diamond chip with a near-surface layer of a high sensitivity NVs where wide-field optical images are taken using a complementary metal-oxide-semiconductor (CMOS) camera. The microwave signal is delivered by a microwire on the diamond, and optical excitation is achieved in total reflection mode. Bright-field images, a confocal image of dye-stained cells together with magnetic images using NV in the bulk diamond, were compared using the same microscope. Here, the diamond quantum microscope is highly simplified to allow a wide field-of-view. To prove the efficacy of the method, it was possible to quantify and show cancer biomarkers corresponding to rare tumor cells within a large population of healthy cells.

NMR spectroscopy using NV centers in diamond consists of advancing NV magnetometry methods to sense the Larmor frequency of nuclear spins close by to the NV sensors. The conventional coil-based induction NMR method has a low detection sensitivity preventing its application to samples at the nanometer scale, while due to the atomic size of NV sensors, coupling at the nanometer scale with nuclei is much more achievable. However, the coupling efficiency depends on the distance, and NV centers must be 20 nm from the nuclei. A single NV created in a ^12^C-enriched diamond layer at 20 nm from the surface was used in [73] to sense protons in the PMMA polymer layer on the surface of a bulk diamond used as a substrate. The proton spins were manipulated by a radiofrequency π pulse (RF) with variable frequency in the presence of a small magnetic field (80 mT), the NV spin being manipulated using a spin-echo sequence. The NMR proton Larmor frequency is measured by a dip in the relative NV spin echo response as a function of the RF frequency. A shift of the measured Larmor frequency is seen in the spin-echo signal by varying the external magnetic field as expected, based on the proton’s gyromagnetic ratio. To improve the spectral resolution, a Fourier transform technique is used. Here a time-domain free induction decay (FID) is obtained by changing the RF π pulse into two π/2 pulses with a time delay τ*n* which is incremented. As the pulse spacing τ*n* is scanned, the NV spin echo amplitude oscillates with a period that matches the proton Larmor frequency and by applying a cosine transform to the time domain data, the frequency spectrum is obtained with a peak in the Larmor frequency.

A different spin sequence is applied in [74] to single NVs in bulk diamond and an NV ensemble imaging modality to sense the Larmor frequencies of H, F and P nuclei close to the NV sensors. In his case, the NV is polarized by the excitation laser in the ground state spin |0⟩. It is then placed in a superposition of |1⟩ and |0⟩ spin states. An XY8-k sequence allows the NV spins to probe the local magnetic environment, and finally, a microwave π/2 pulse projects the evolved NV spin coherence onto a |0⟩, |1⟩ state population difference. The XY8-k sequence serves to extend NV spin coherence times, and as a spectral filter, to increase the sensitivity to specific nuclear spins. It consists of a k-times repeated sequence of eight π pulses, with a delay time of τ between π pulses, which is related to the frequency of the sensitivity that is enhanced. By changing τ, different nuclear spin frequencies are probed.

The first 2D imaging of nuclear spin was reported in [75]. Here an XY8-96 sequence is applied to a single NV center in ^12^C-enriched diamond to provide a two-dimensional image of ^1^H NMR from a polymer test sample with a spatial resolution of ≈12 nm.

Towards NV-NMR imaging, NV-NMR was used in [76] to determine the accurate position of individual ^13^C nuclear spins near the NV sensor within the diamond by nuclear resonance spectroscopy experiments. The three-dimensional spatial coordinates of the nuclear spins are converted in a 3D image with sub-Ångstrom resolution and for distances beyond 10 Å. Here a single NV center close to the diamond surface is used to sense the precession of nuclear spins under a rapidly switching magnetic field obtained with a coil on the diamond surface. To retrieve the azimuth angle of the nuclear spins and reconstruct a 3D image, the authors achieved a dynamic tilt of the quantization axes, induced by a transverse variation of the magnetic field generated by a high-bandwidth microcoil together with high-resolution correlation spectroscopy. Four ^13^C atoms were localized in a 3D map compared to NV. It is accepted that the prospect of a general single-molecule MRI technique has some challenges. These include isolating a single molecule in a spin-free matrix layer, suppressing nuclear dipolar interactions and reducing line widths and spectral complexity.

A method is proposed in [77] to engineer nuclear spins within the diamond layer consisting of ^13^C nuclear spins doped with NV embedded in a spin-free ^12^C crystal matrix. In this case, a few tens of nuclear spins are controlled by using radio frequency pulses, and it is shown that nuclei spin coherent control such as Rabi oscillations and Ramsey spectroscopy is possible.

**Figure 4:**
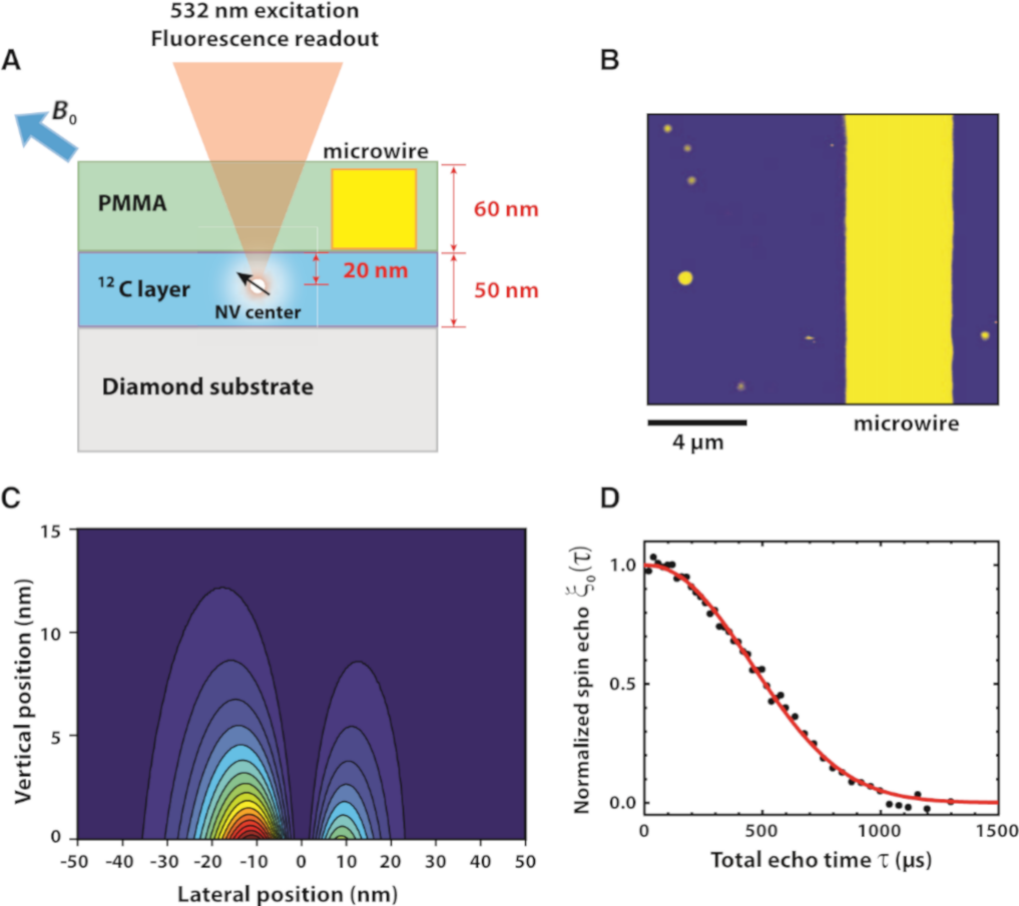
NV-NMR detection system schematic of [73]. Image reprinted with permission from [73], copyright 2013, AAAS. (A) ^12^C diamond layer with NV center spin at [111] orientation embedded at 20 nm depth. Proton NMR is detected in a PMMA layer. (B) Sample surface from fluorescence imaging, with microwire and NV center (circled). (C) Proton detection sensitivity dependence from space along the PMMA layer cross-section. 50% of the proton signal comes from a (24 nm)^3^ volume. The NV center axis is 54.7° tilted from surface normal, originating two lobes as shown. (D) Normalized spin echo response vs total echo time.

Figure 4 presents the NV center nano-MRI set-up of [73]. This is one of the first attempts to detect proton NMR in an organic sample facing the diamond by using an individual NV center close to the surface. Electron spin echoes and proton spin manipulation are combined so that the NV center senses the nanotesla proton field fluctuations, allowing simultaneous nanometer scale time-domain and spectroscopic NMR measurements.

NMR spectroscopy of organic/biological material is possible only for samples of significant size [78]. Single-molecule ubiquitin-protein NMR sensing at room temperature is possible by connecting them to a diamond surface that has been functionalized with pairs of qubits whose interaction mimics the coupling of an electronic spin with an ancillary nuclear spin. The sensitivity can thus be improved to the point that single proton spins become detectable in integration windows as short as 1 s, as the diamond surface is treated to improve the spin coherence of the NV centers and detection accuracy benefits from the quantum logic relationship between the qubits. The spectral fingerprint obtained allows recognition of single proteins and their chemical composition.

The microwave RF power needed to create a singlet state is high enough to pose safety issues for in vivo imaging of biomaterials. However, recent experiments show that a long lifetime nuclear spin-singlet state can also be created by RF spin-locking fields at powers that are two orders of magnitude lower than the conventional method. These states also receive help from a long singlet-triplet coherence time that persists even after the excitation is removed [45].

Improving the contrast mechanism is another important research avenue for MRI and NMR as recently proven. Suppression of undesired chemicals using the contrast-enhancing singlet states (SUCCESS) based on long lifetime nuclear singlet states supplies an enhanced in vitro capability for resolution of hard to detect peaks in the background noise. In this mechanism, singlet states are originated in the target molecule and stabilized by a continuous RF emission to create a quantum filter. Subsequently, after they have affected the magnetic saturation of the undesired molecules, they are transported back to the earlier state by a specific pulse sequence and detected [46].

Shallow NV centers have been exploited to effect multiple-species MRI and bulk NMR spectroscopy at field intensities two orders of magnitude lower than traditional methods, achieving room-temperature nanoscale imaging of proton spin ensembles for fields as low as 20 mT [65].

To improve the imaging resolution, it is important to accurately measure the NV center depth under the surface of the diamond. One recently proposed method combines confocal and NMR imaging of NV centers and modeling the polarization statistics of proton spin interactions, reaching a resolution of 1 nm and achieving good matching with ion implantation simulations [79].

**Figure 5:**
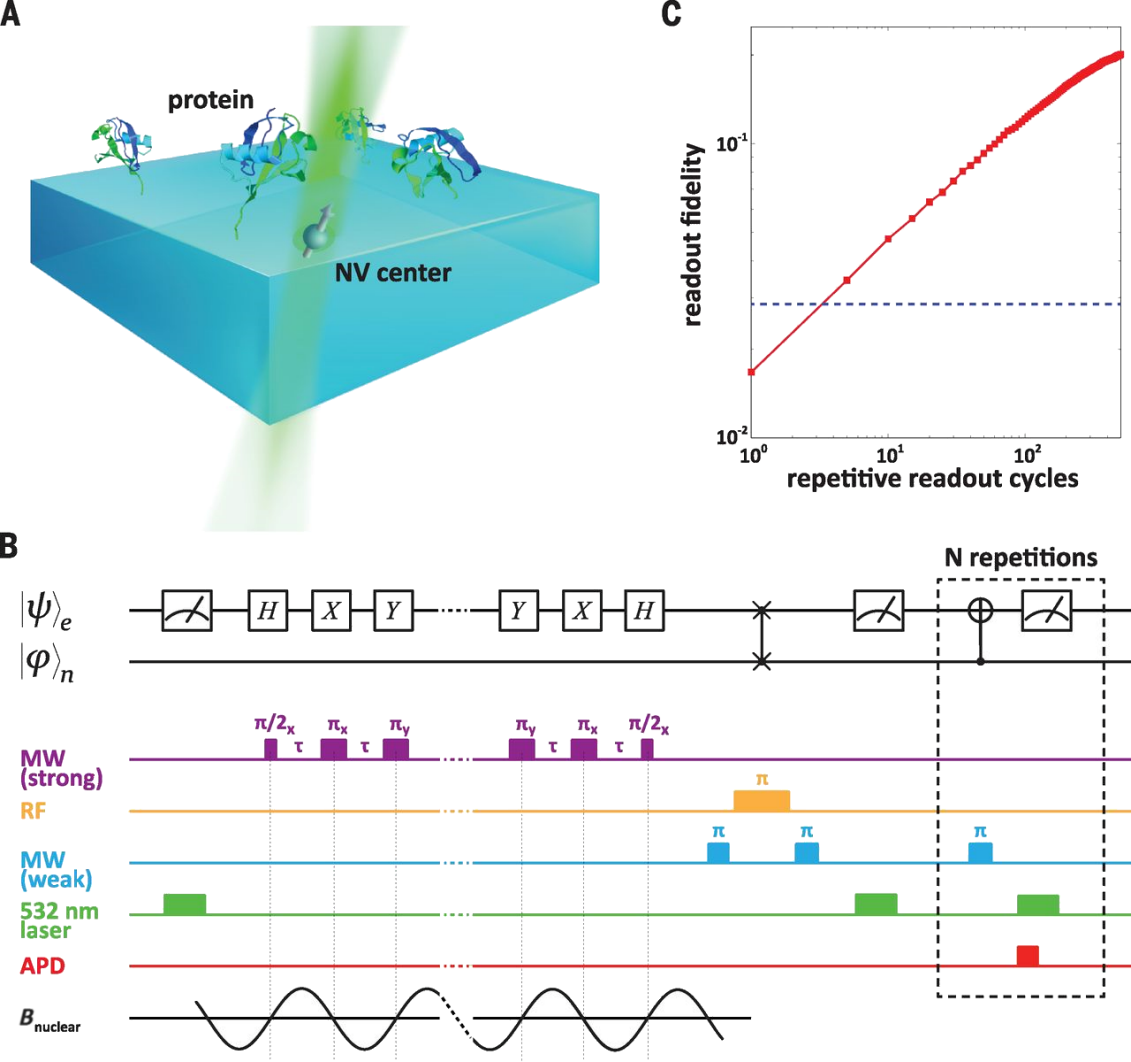
Experimental setup and magnetometry with repetitive readout [78]. Image reprinted with permission from [78], copyright 2016 AAAS. (A) Experimental setup. NV center electronic spin and its associated 15 N nuclear spin are used as a proximal quantum sensor to probe ubiquitin proteins on the surface of the diamond. (B) Quantum circuit diagram and experimental magnetometry pulse sequence. (C) Repetitive readout cycles and measured fidelity gain. MW – microwave; RF – radiofrequency; APD – avalanche photodiode; B – nuclear spin magnetic field.

Figure 5 shows the layout of a repeated detection magnetometry experiment from [78], aiming for single-protein spectroscopy and NMR. A statistically polarized subset of proximal protein nuclear spins produces a time-varying magnetic field whose single Fourier component is measured. Precession at the nuclear Larmor frequency affects the spin ensemble transverse magnetization, giving stochastic variations in phase and amplitude at each sequence repetition. The MR signal is obtained by averaging over many iterations, which gives a zero-mean magnetization with nonzero variance. NV center sensing is carried out by manipulating the spin state by a periodic sequence of microwave pulses separated by free-evolution intervals of length τ. Narrow band-pass filtering is affected by the periodic modulation of the NV center spin, which accumulates the phase when the modulation frequency, defined as 1/τ, is close to twice the nuclear Larmor frequency. The interval variation between the π pulses encodes the protein nuclear spin information within the measured frequency spectrum. High resolution in both space and time have been looked for with various other setups of NV center sensors.

NV center magnetometry can measure the tiny magnetic fields produced by neural action potentials, allowing for in vivo neuron imaging. Neural complex dynamics measurement requires imaging at subcellular or synapse-scale resolution in order to image single neuron dynamics in living tissue. Typical neural pulse durations are around 2 ms with a magnetic field of ≤10 nT and peaks at 100 nm from the axon, which is challenging for NV center magnetometry. By considering an ensemble of NV centers in a single-crystal ultrapure diamond membrane, a model simulating the time dynamics of the axon has been developed, while also considering a detection system relying on wide-field NV center photoluminescence imaging in a confocal microscope. Options for magnetic field detection include continuous ODMR and free induction decay, with sensitivity in both cases of 10 μT·Hz^−1/2^. The specific sequence repetition and applied sensing volume (1 μm^3^) necessary to image a single axon depends on the size of the axon.

Single-neuron label-free imaging is possible with current NV-magnetometry techniques at a 10 nm spatial resolution, 30 μs temporal resolution and 1 mm field-of-view without the invasiveness and toxicity in vivo, allowing for long observation periods using single-crystal diamonds supporting the sample on a uniform 13 μm surface layer of high-density (3 × 10^7^ cm^−3^) NV centers. The temporal detection of the NV center MR frequency due to the action potential magnetic field is available with a temporal resolution of 32 µs and a magnetic field sensitivity of 15 pT·Hz^−1/2^, giving a single-neuron measured the magnetic field of ≈4 nT peak-to-peak.

In order for biomedical imaging to be applied to processes with a wide range of length scales (from micrometer to nanometer) and to be used to investigate biological functionality down to the electron configuration of single proteins and DNA sequences, nano-MRI is required, which should also be capable of imaging internal dynamics. Strong magnetic pulses are used in conventional MRI, however, at very small scales greater sensitivity is needed instead. Molecular MRI needs sensors and optical probes on the scale of a single atomic spin [12].

The application of nano-MRI with diamond NV centers is possible due to NV spins having magnetic dipole–dipole interactions with other spins close by, which does not need the usual high MRI magnetic fields and obtains high SNR wideband signals at nanometer resolution. NMR imaging of groups of polymer nuclear spins is proposed in optical confocal and wide-field microscopy by increasing the complexity of microwave pulse sequences to stabilize (with respect to frequency) the coherence time of NV center spins, and also using scanned magnetic probes functionalized with nuclear spins for nanometer resolution 2D proton imaging and even tighter 3D imaging of dark electronic spins [12].

NDs of less than 10 nm diameter can be employed as NMR sensors due to the atomic size and photostable fluorescence of the NV centers, whose electron spins can be managed by microwave pulse sequences and detected by observing luminescence. Ultrapure bulk diamond grown isotopically at room temperature allows for millisecond-scale dephasing using Hahn-echo pulse sequences, while dynamic decoupling pulse sequences at 77 K make it possible to reach a 1 s coherence time. The NV center excited triplet state is optically pumped via a dark-state spin-dependent transition and couples to the magnetic field by a spin-orbit effect that is mediated by transitions triggered through an external microwave signal. Thus, the fluorescence is amplitude-modulated by the magnetic field with a positional accuracy given by the NV center size. The reported sensitivity is around 40 nT·Hz^−1/2^ for DC (direct current) magnetometry and around 10 nT·Hz^−1/2^ for AC (alternating current) magnetometry. MRI of single nuclear spins is limited by the weakness of their interaction with NV center spins, however, single NV centers can have a resolution down to 12 nm when imaging ^1^H NMR in polymers [12].

Super-resolution techniques such as stimulated depletion emission microscopy (STED) and stochastic optical reconstruction microscopy (STORM) are fully optical methods that can deliver nanometer spatial NV center resolution, thus giving rise to methods that combine STED and STORM with spin resonance methods based on spin-echo sequences. For instance, Fourier magnetic imaging (FMI) is based on NV center spin Fourier (or k-space) phase encoding for magnetometry. A 3.5 nm resolution is achieved by FMI imaging in the k-vector space, and phase encoding of spatial information on NV center electronic spins in the “k-space” is obtained by pulsed magnetic field gradients. After the wavenumber measurement, the image is inverse transformed in the real space by fast Fourier transform (FFT), giving a wide-field view at a short computational time. This has the advantage of space multiplexing with high SNR and acquisition rate, as the methods work by compressing the sensing in wavenumber space and simultaneously reads out all the NV centers within the field-of-view [30,80].

Spin-hosting, NV color centers in diamond, have the sensitivity in space to detect NMR signals down to single molecules. However, the resolution in time (frequency) of present schemes is insufficient to resolve the details of molecular structures. Recently, an NMR scheme exploiting an NV center ensemble used a synchronous readout technique which enables the frequency resolution to be set independent of the coherence time of the sensor. Diffusion effects are mitigated by the wider volume of sensing and allow the thermal, rather than statistical, nuclear polarization to be accessed. The technique achieves a ≈3 Hz spectral resolution, allowing chemical shifts and nuclear spin–spin couplings to be resolved.

The NV centers are atomic-size defects isolated from the surroundings and act as atomic-scale magnets with optical photostable emission. As they are embedded in diamond matrix they are biocompatible and can be regarded as atomic size probes. These probes can be in close proximity or introduced into living cells and tissues. NV center detection of very weak magnetic fields is done via their light emission modulation by a local magnetic field. Due to their size they are used as ultrasensitive magnetic probes to monitor the local magnetic field strength and direction changes over sub-micrometer distances.

NV centers in diamond have already been used to record NMR signals from samples on the nanometer scale. For example, enough sensitivity to detect a single protein magnetic field was shown in [78]. However, the best resolution in frequency for NMR of molecules has been only about 100 Hz [81]. Applied to nanometer and micrometer volumes, NV center protocols produced broad NMR spectral lines of more than 100 Hz. This is the result of both the short spin state lifetime of the NV center, of about 3 ms, in addition to the fluctuating statistical spin polarization of the sample [82]. This frequency is not enough to resolve key spectral features of molecular structures that are critical to many conventional NMR applications. The inductively detected conventional NMR has the necessary high spectral resolution, but it has limited sensitivity in space, necessitating samples on the millimeter scale.

A sensitive magnetometer based on an ensemble of NV centers can deliver the sought spectral resolution down to about 1 Hz [82]. The readout protocol is an evolution of the work in [83–84]. The spin sensor is an ensemble of NV centers in combination with a narrowband synchronized readout protocol to obtain the NMR spectral resolution of about 1 Hz. The samples are of micrometer scale and allow for thermal, rather than statistical, nuclear polarization to be obtained at a spectral resolution of about 3 Hz. This resolution permits chemical shifts and nuclear spin–spin couplings to be resolved [82].

This protocol is used to sense NMR signals for as long as 103 seconds. By achieving an NMR spectral resolution of about 1 Hz in the sample volume of a typical cell, it is possible to observe key spectral features that would otherwise not be detectable.

**Figure 6:**
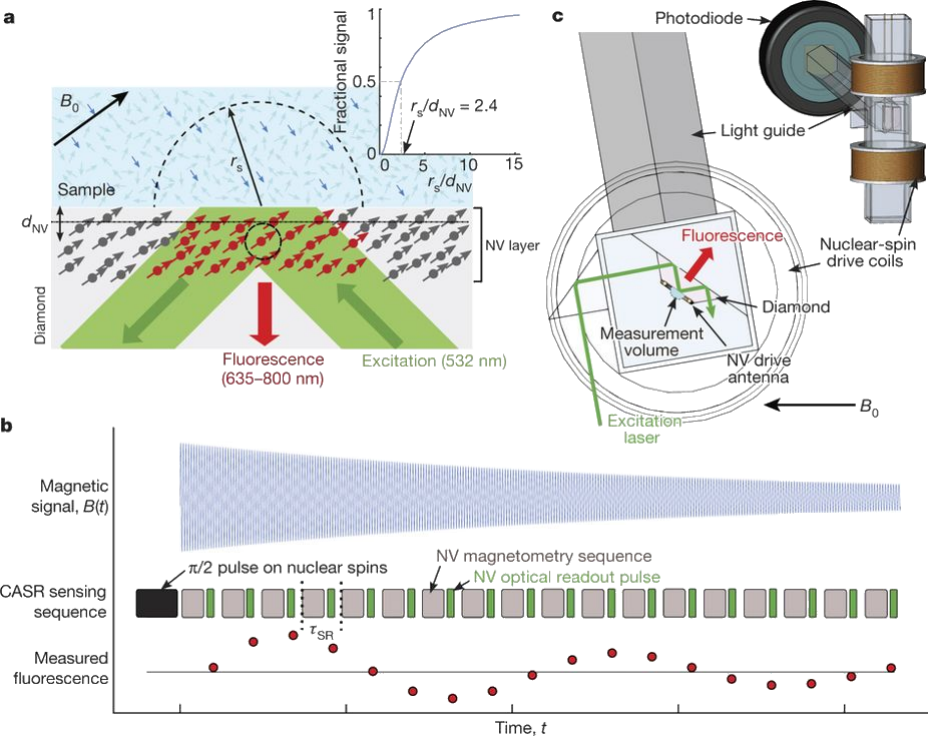
NV-ensemble sensor for coherently averaged synchronized readout (CASR) NMR. Image reprinted with permission from [82], copyright 2018, Springer Nature Publishing. (a) NV-ensemble sensor based on an NV center surface layer on a diamond chip, probed by a green laser. Thermally polarized nuclear spins are NMR detected from the sample. (b) CASR detection of an NMR FNP signal. Top row: thermally polarized spin precession at frequency *f* causes oscillations in the magnetic field. Middle row: CASR protocol made of optical NV spin-state readouts blocks repeated at the synchronized readout cycle period intertwined with blocks of identical NV AC magnetometry pulse sequences with central frequency. (c) Probe geometry: a cuvette holds the sample and the diamond chip, above and below are the cylindrical coils driving the nuclear spins, while a wire antenna drives the NV centers. A light guide collects the spin-state-dependent fluorescence from the NV to bring it to a photodiode, while an electromagnet supplies the magnetic bias field.

The measurement technique of [82] is presented in Figure 6. This NMR scheme [82] enables frequency resolution independent of the sensor coherence time [85]. This approach seems superior to alternatives such as in [81,86–87].

The intrinsic nitrogen nuclear spin was used in [86] and [87], as a longer-term memory for the first phase measured in correlation spectroscopy with extended interrogation times. Repetitive, non-demolition nuclear state readouts are integrated into this approach that also allow decoupling of the sensor electron spin back-action on the target nuclei, thus shortening their correlation time [87]. Sample diffusion here limits the sensing duration (≈5 ms). However, the nuclear memory approach resolved ppm-level chemical shifts in liquid-state samples [81].

In the sample application in [82], the technique was used to observe NMR couplings under ambient conditions in a micrometer-scale sample volume of about 10 picolitres. NMR is applied to thermally polarized nuclear spins of an ensemble of NV centers to resolve chemical shift spectra from small molecules, allowing full chemical specificity of a single biological cell. At sampling frequencies down to 1 Hz, the new measurement scheme is 100× the spectral resolution of prior NMR spectroscopy based on NV centers.

The scheme presented in [82] has the potential to apply NMR spectroscopy down to the single-cell scale. Further developments are still needed to boost the signal from small samples to make it faster and more applicable to living samples, as well as to improve the sensitivity of the NV centers to detect faint signals produced by samples in weak concentrations.

If it were possible to have a certain type of color centers demonstrating performance comparable to quantum probes for NV centers in diamond, quantum sensors based on SiC would be much improved. However, so far, no color centers in SiC have shown promise as quantum sensors at room temperature. The optical contrast of the spin signal, divacancy and silicon-vacancy in SiC is limited to below 10% at room temperature, and the overall count rate is also lower than that of the NV in diamond. Thus, solid-state spin sensing MR spectroscopy continues to progress in diamonds.

The length of time for which phase is accumulated limits precision in quantum sensing applications and quantum memory has been employed to increase precision by extending this time to longer than the coherence lifetime. The use of a quantum memory allows for increased sensitivity as well [86], employing entanglement in a hybrid spin system based on a single NV center linked to sensing and a memory qubit. In this way the quantum state is preserved beyond the coherence decay, allowing for coherent interaction with distinct weakly coupled nuclear spin qubits. The performance is measured by the stepped increase of entanglement between sensor and memory, which is compared with the performance of the lone sensing qubit, looking forward to high-resolution NMR spectroscopy of single ^13^C nuclear spins.

Additionally, sensor dissipation limits the measurement performance at the nanoscale, reducing the sensitivity and even causing relaxation or dephasing. At room temperature, weak NV center dissipation allows individual target nuclear spins to be detected but limits the spectral resolution to several hundreds of Hz, which is too low for molecular recognition. NV intrinsic nuclear spins can be used as static memory for NMR [87] and avoid back-action of the sensor on target by controlled decoupling of the sensor, memory, and target. This increases the memory lifetime up to 4 minutes, enough to apply efficient measurement and decoupling protocols, while achieving a room-temperature resolution of 13 Hz.

Environmentally mediated resonance (EMR) control of spin qubits is considered in [88]. Magnetic field sensitivity and maximum field range are difficult to optimize at the same time. However, interferometry-based magnetometry, based on quantum two-level systems (which dynamically vary their phase according to the magnetic field) works to address the issue [89]. Since the phase is 2π periodic, an increase of the coherent interrogation time to improve sensitivity causes a reduction of the field range. The measurement of the geometric phase in a quantum two-level system introduces a way to address this, that is, by employing the diamond NV center electronic spin, which allows unwrapping of the 2π phase ambiguity with a 400× increase in range. The nonadiabatic regime affords an added increase in sensitivity and significantly affects phase decoherence.

Back-action in measurements can be addressed by protecting the spin state through multilevel ^14^N nuclear-spin memory, allowing for repeated readouts and making it possible to use error correction based on the quantum logic of coherent feedback to reverse measurement back-action. This results in a 13-fold enhancement of readout fidelity and a 2-fold improvement in the signal [90].

Robust estimation methods can aid in the measurement of spectral properties in classical and quantum dephasing environments through filter function orthogonalization, optimal control filters maximizing the relevant Fisher Information, and multi-qubit entanglement [91]. Scheme robustness under noise is considered, looking at effects of finite-precision measurements, dephasing of the probe, spectral leakage, and slow temporal fluctuations of the spectrum.

While the above-described approaches using NDs in hyperpolarization are not related to the presence of NVs in the NDs, but simply try to use the material with conventional MRI methods using paramagnetic electrons spin, other approaches are followed by combining efficient DNP with optically polarized NV. These approaches are more relevant in the context of NV nano-MRI applications, even if they appear difficult for scale-up purposes and none of the current realizations of polarization transfer are simultaneously robust and efficient. In [92], a fast and robust approach to achieve DNP of NV polarization to ^13^C nearby nuclear spins is proposed and experimentally demonstrated in single NV centers in a diamond. The design of sequences of short pulses with longer waiting periods between the pulses achieves a polarization transfer through many repetitions of the sequence. This method can be applied for the polarization of nuclear spins of molecules external to the diamond using an ensemble of close-to-surface NV centers or for the hyperpolarization of ND as MRI biomarkers. This hyperpolarization method is much simpler than the others described above, mutated from MRI technologies, as it can be achieved at room temperature.

The application of the above method to NDs is still challenging as NVs in different NDs have different spin resonance properties due to the random lattice orientation of the NDs to the magnetic field orientation. This problem is similar to the application of the ODMR-based magnetometer using NDs rather than an ensemble of NVs in bulk diamond, as it is hard to collectively polarize the NVs in all NDs at the same time. An attempt to solve this problem is reported in [93]. Here, to overcome the challenges to optically hyperpolarize diamond powder, a DNC sequence has been applied to 200 μm diamond particles. The polarization transfer is achieved by optically pumping NV centers to ^13^C in diamond particles and by a first microwave irradiation at low field values (1 to 30 mT), after which the sample is subject to rapid bulk inductive readouts at 7 T. The process occurs at room temperature; however, the relaxation time appears shorter to that obtained by using brute force in large diamond crystals. Overall the combination of optical polarization of NV seems to improve ^13^C polarization by orders of magnitude compared to those obtained using thermally polarized P1 centers under comparable conditions [19]. However, the transfer to biomedical imaging using conventional MRI with these particles sizes is a challenge.

Detecting and controlling nuclear spin nano-ensembles is crucial for the further development of NMR spectroscopy. The fabrication of mono-atomic layers of nuclear spins and their control is achieved in diamond NV centers in [77]. Using chemical vapor deposition, a nanometer-thick diamond layer of ^13^C carbon atoms was grown in between two layers of a ^12^C-enriched diamond (acting as spin dephasing protecting caps), on an ultrapure diamond substrate. The ^13^C layer was doped with nitrogen via δ-doping during the growth process to create a single NV near the ^13^C layer. In another sample, the NV was implanted at different depths within the ^13^C layer. In both samples, single NVs are coupled to a few tens of nuclear spins, thus enabling polarization and readout of the magnetization of these small ensembles. This work has principal applications in quantum simulation with an engineered quantum Hamiltonian. A fabrication method is provided, which is a useful tool to improve nuclear spin polarization via optical NV electron spin polarization.

A new method has been proposed in [94] to hyperpolarize surrounding nuclear spins (hydrogen nuclear spin ensemble in molecular poly(methyl methacrylate)) on the diamond surface using NVs in a diamond probe at room temperature without resorting to any radio-frequency or microwave sequence. An external low magnetic field is used to tune the ground-state spin transition frequency of the NV into resonance with target nuclear spins. The NV is initially optically polarized and readout is performed via its photoluminescence signal. While an extended MRI contrast agent hyperpolarization small chamber is proposed, this method appears challenging for its scale-up potential in biomedical applications. Considering the many recent contributions to achieve hyperpolarized NDs for conventional MRI-aided (or otherwise by NV optical polarization and microwave sequence), this topic appears to be a growing area of research interest. However, the path to move to the use of NDs as MRI contrast agents with or without NV centers is still quite long.

NMR sensors based on optically probed NV defects in diamond have allowed molecular spectroscopy from sample volumes that are several orders of magnitude smaller than the most sensitive inductive detectors. However, NV-NMR spectrometers have only been able to observe signals from pure, highly concentrated samples.

To advance the applications in mass-limited chemical analysis and single-cell biology using NV-NMR, it is necessary to increase the spectral resolution and the concentration sensitivity. For this purpose, the use of diamond quantum sensors as in-line microfluidic NMR detectors is presented in [95], where by spatially separating the polarization and detection phases in a microfluidic platform, picoliter-volume sensitivity is achieved. Two-dimensional correlation spectroscopy within ≈40 picoliter detection volume is performed with a spectral resolution of 0.65 ± 0.05 Hz, which is an improvement by an order-of-magnitude over the earlier diamond NMR study.

Finally, by combining picoliter-scale NV-NMR that uses a CASR NMR protocol with 1 Hz resolution with Overhauser dynamic nuclear polarization (DNP) using electronic spins in the TEMPOL radicals, high-resolution spectroscopy on a variety of small molecules in dilute solution was achieved reaching femtomole sensitivity [96].

### Conclusion

This review outlines many of the nano-MRI techniques that are currently under development. It is arguable that NV centers in diamond are one of the most promising of these techniques. In this review, we have summarized several methods that use NV centers in diamond for nano-MRI involving magnetometry at the nanoscale. These are based on the NV sensor paramagnetic resonance properties that provide sensitivities compatible with nano-MRI for nuclear spins.

The research field presents a variety of material sensors such as NV in bulk diamond structured from single NV to large ensembles of NVs (layers of many NVs under the diamond surface), fabricated arrays of NVs below the surface, and in NDs as ensemble-producing FNDs. The current best sensitivity is achieved using an ensemble of NVs in a layer in bulk diamond. However, the use of arrays of high-density NVs is a very desirable approach for multichannel magnetic sensing.

The use of NDs for nano-MRI is still extremely limited, mostly due to the lack of high-quality NDs, and few examples of applications of NDs as temperature and turbulence fluid sensors have been shown. The functionalization of NDs with magnetic contrast agents such as MNPs and Gd is another area of interest for applications for nano-MRI. Similarly, attempts at hyperpolarization of NDs with and without NV centers have been performed with no applications in biomedical studies developed to date due to the limited contrast.

In terms of methods, we have found many approaches that lead to different sensitivity and spatial resolution. The highest sensitivity and spatial resolution are not always achieved in the same experiments due to the excessive complexity associated with the combination of both in the same setup/protocols.

The simplest approach is NV ODMR magnetometry, which can reach sensitivities as low as nT·Hz^−1/2^ in bulk diamond (100 nT·Hz^−1/2^ in NDs) and has been demonstrated in many applications in fields of biomedical imaging and condensed matter physics. Some remarkable biomedical applications include imaging magnetotactic bacteria with subcellular resolution (400 nm) with a micrometer field-of-view, imaging mammalian cells with nT sensitivity and sub-micrometer resolution, sensing neuron action potentials with single-neuron sensitivity, optomagnetic imaging of neural network activity in brain slices, and 3D magnetic imaging of macrophage labeled with superparamagnetic iron oxide nanoparticles for liver tissues specimens. These last specific applications are currently addressing biomedical needs and are likely to achieve further development due to the similarity to conventional MRI applications with enhanced sensitivity and resolution. They are, however, based on sensing of magnetic contrast agents or magnetic biological systems rather than nuclear spins within these systems.

ODMR-based magnetometry is also used for solid-state physics metrology in thin film ferromagnetic materials, current distribution in 2D layer materials, magnetic noise, and superconductor critical field applications. Advanced ODMR magnetometry has been combined with super-resolution microscopy methods, such as STED and STORM, achieving spin localization with high spatial resolution of up to 20 nm, even in NDs.

More complex NV magnetometry radiofrequency and microwave sequences have been developed for NMR with a sensitivity of a few pT·Hz^−1/2^, which is the closest approach to conventional MRI while using NV as a sensor. Many different approaches are used for NV NMR. However, small ensembles of nuclear spins in polymer spectroscopy and single protein nuclear spectroscopy were achieved by NV centers in bulk diamond. NMR spectroscopy has been converted to the 2D imaging of a dilute ensemble of ^1^H with 12 nm resolution. A 3D image of nuclear spins of NV in near proximity to ^13^C atoms has been achieved with a sub-nanometer resolution; however, 3D imaging of single-molecule nuclear spins still remains a challenge.

High sensitivity AC NV magnetometry has been combined with super-resolution microscopy methods to increase the imaging resolution. The frequency resolution of NV center NMR is limited to 100 Hz, due to the broad nuclear spin resonance detected from single proteins, while conventional NMR has a high spectral resolution. This problem has recently been resolved by implementing a CASR NMR protocol, reaching 1 Hz frequency resolution and providing the ability to sense molecular structures critical for conventional inductive NMR.

In general, a great deal of progress has been made using simple ODMR NV magnetometry which recently has shown great potential in biomedical applications. However, this method cannot be applied to any biological system but only to specific cases.

Regarding NV NMR, which paves the way for a truly nano-MRI general method, the main challenge is to achieve 3D imaging of nuclear spins in molecules and develop algorithms and protocols to achieve this aim. The sensitivity should also be improved to fT·Hz^−1/2^. In regard to the development of protocols to achieve the required sensitivity for 3D imaging of nuclear spins at the single molecular level, a road map should be generated to fast track outstanding applications. It is understood that one of the main challenges that remains is the isolation of single molecules in the spin-free layer.
